# Detection of Occluded Small Commodities Based on Feature Enhancement under Super-Resolution

**DOI:** 10.3390/s23052439

**Published:** 2023-02-22

**Authors:** Haonan Dong, Kai Xie, An Xie, Chang Wen, Jianbiao He, Wei Zhang, Dajiang Yi, Sheng Yang

**Affiliations:** 1 School of Electronic Information, Yangtze University, Jingzhou 434023, China; 2 Western Research Institute, Yangtze University, Karamay 834000, China; 3 School of Computer Science, Central South University, Changsha 410083, China; 4 National Super-Computer Center in Changsha, Hunan University, Changsha 410082, China; 5 School of Information Science and Engineering, Hunan University, Changsha 410082, China

**Keywords:** image super-resolution, occlusion small commodity detection, residual dense block, attention mechanism, feature pyramid network, feature enhancement

## Abstract

As small commodity features are often few in number and easily occluded by hands, the overall detection accuracy is low, and small commodity detection is still a great challenge. Therefore, in this study, a new algorithm for occlusion detection is proposed. Firstly, a super-resolution algorithm with an outline feature extraction module is used to process the input video frames to restore high-frequency details, such as the contours and textures of the commodities. Next, residual dense networks are used for feature extraction, and the network is guided to extract commodity feature information under the effects of an attention mechanism. As small commodity features are easily ignored by the network, a new local adaptive feature enhancement module is designed to enhance the regional commodity features in the shallow feature map to enhance the expression of the small commodity feature information. Finally, a small commodity detection box is generated through the regional regression network to complete the small commodity detection task. Compared to RetinaNet, the F1-score improved by 2.6%, and the mean average precision improved by 2.45%. The experimental results reveal that the proposed method can effectively enhance the expressions of the salient features of small commodities and further improve the detection accuracy for small commodities.

## 1. Introduction

In recent years, owing to the continuous development of technologies such as big data and the Internet of Things, artificial intelligence has gradually matured. The government has issued relevant policies to support the transformation of the retail industry to digital platforms and further promote the development of an intelligent retail industry; consequently, the offline retail market has witnessed continuous expansion. The global retail industry market size reached 27 trillion USD in 2021, and as per estimates, artificial intelligence will contribute additional growth of 2 trillion USD to retail by 2035, thereby providing massive business value.

Currently, two main solutions exist for retail containers: non-visual and visual methods. Non-visual methods primarily include gravity sensing and radio frequency identification technologies. However, these methods exhibit poor flexibility and increase the cost of commodities. At present, numerous target detection methods are based on convolutional neural networks (CNNs), such as faster-region-based CNNs (Faster-RCNN) [1], single-shot detection (SSD) [2], YOLO [3], and RetinaNet [4]. Retail containers in the market are increasingly using visual container technology [5] based on deep learning for commodity detection [6,7] and identification to realize the deduction of commodities purchased by customers and corresponding settlements.

Owing to the influence of light, transmission equipment, and the surrounding environment, the details of a video image can be substantially lost. Some researchers have conducted studies related to image super-resolution (SR) [8] to solve the problem of image blurring. For example, Noh divided an input low-resolution image into textured and non-textured regions [9] and then interpolated the image according to the features of local structures, thereby retaining the texture and structure information of the image while ignoring the contour information. To reduce the number of parameters and ensure good performance of the network, an ultra-lightweight SR network [10] was proposed to retain high-frequency details. However, the restored images exhibit structural distortions. Therefore, Ma [11,12] proposed a structure-preserving SR method with gradient guidance to alleviate the geometric distortion prevalent in the SR results from perception-driven methods. Additionally, a new module called feature texture transfer (FTT) [13] was used to extract trusted regional details. A texture- and detail-preserving network [14] was proposed, which can not only learn local and regional features but also pay attention to texture and detail features and restore high-resolution ratio images with better perceptual effects. In addition, some experts [15] decoupled the reference-based super-resolution from a new perspective, eliminating the interference between the LR image and the reference image. However, the generated image is easily lacking constraints with the original image. In this article, we combine texture, content, and contour features to obtain rich SR image information.

Recently, in terms of feature extraction, CNNs [16], residual networks [17,18], and other networks have been used to extract the target features. As small commodities are occluded [19], the effective features of such commodities are often missing, so image inpainting [20] algorithms are usually used to repair the incomplete image. However, existing studies can only display excellent results in accomplishing simple image structures and generating image content with a complex overall structure, and high fidelity of detail remains a huge challenge. Therefore, optimized residual mapping [21] was used to improve the learning ability of the residual network. Zhang [22] used densely connected convolution layers in residual dense blocks to extract rich local features. They reported that stacking additional residual blocks enhances the normalization preservation of a network [23]. Although these networks perform well at extracting features, they are extremely complex, resulting in a significant loss of efficiency. Some scholars [24,25] have adopted residual learning to gradually improve by learning the residual in each output, which can be achieved with only a few convolution parameters, thereby achieving high compactness and efficiency. A novel squeeze-and-excitation module (SENet) [26] was proposed. This attention mechanism focuses on each input channel, and the network focuses on the important channel after obtaining the weight of the corresponding channel, thus significantly improving the performance of the CNN. Wang [27] designed an efficient channel attention module to significantly improve model performance while using fewer parameters. Liu [28] proposed a pixel-level context attention network for selectively focusing on the context location information of pixels and generating an attention force to generate the context features of salient targets. Compared to SENet [26], which focuses only on the attention mechanism of the channel, Woo [29] conceived a lightweight convolutional attention module (CBAM). This module infers an attention map from the channel and spatial dimensions in turn and outputs refined features. Instead of simply using the residual network to perform feature extraction, we add the attention module based on this, which makes the network pay more attention to the detailed features of commodity regions, fully extracts the spatial information of multi-scale feature maps, and realizes the interaction between important features of cross-dimensional channels and spatial attention.

In the process of target prediction, small targets are easily ignored by the network because of their relatively few features [30]. The detection effect for small targets can be significantly improved by enhancing their features. However, the accuracy of target detection is unstable owing to uncertainty in the features of multiscale fusion. To efficiently express small target features, a new enhanced feature pyramid network (FPN) [31] was proposed, which can suppress redundant semantic information, ensure the enhancement of target features, and significantly improve the detection performance of objects. To improve the detection performance caused by weak features, neighborhood erasing and neighborhood transmission modules [32] were introduced to erase the salient features of large targets and emphasize the features of small and medium targets in shallow layers, respectively. Additionally, recognizing that boundary and texture features help to detect targets, researchers use boundary and texture enhancement networks [33] to embed feature information into object features to predict targets. Wang [34] proposed an “Attentive WaveBlock” module that can be embedded in dual networks to enhance the complementarity between the two parts and further suppress noise.

At present, object detection networks with deep learning as the mainstream are widely used in intelligent retail containers [35], but there is still a lot of room for improvement in the accuracy of commodity detection, especially in the detection of small commodities occluded by hands. There are still problems such as a low detection rate, false detection, and missed detection. Based on this, it is necessary to conduct in-depth and detailed research on the detection of the occlusion of small commodities. In this article, aiming at the detection of small commodities in a smart retail container, especially in the situation where customers’ hands occlude commodities during the purchase process, this paper proposes a feature enhancement occlusion detection algorithm for small commodities with SR. Since the video needs to be compressed and uploaded to the cloud server for corresponding small commodity detection to obtain high-definition images, it is processed with SR, and the corresponding feature expression ability is enhanced to effectively improve the detection performance of small commodities when the features of small commodities are occluded during the detection process.

In summary, this study makes the following three contributions:

(1) During the experiments, we found that the image clarity of the video frames was low; therefore, we processed the images with SR and image super-reconstruction to recover clear images containing more detailed features of commodities;
(2) To obtain more information about commodity features, a convolutional attention mechanism was used to guide the network to extract important features of the commodity while suppressing irrelevant features to fully extract the effective features of the commodity;
(3) As small commodities have fewer features, extracting discriminative features is challenging. Therefore, we enhanced the contour and texture features of the commodity regions to ensure that the features of small commodities could be efficiently expressed and the detection accuracy could be improved.

The structure of this article is as follows: Section 2 describes the overall algorithm and related theories. Section 3 presents the experimental details, including the experimental platform, comparison experiment, ablation experiment, experimental results, and analysis. Section 4 summarizes the proposed algorithm.

## 2. General Architecture

**Figure 1:**
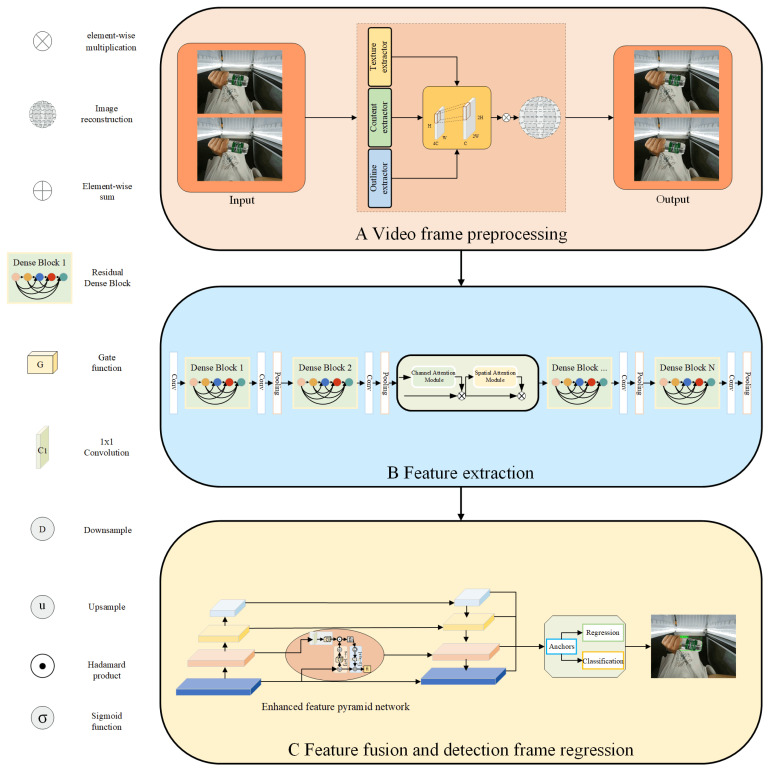
Algorithm flow. (**A**) The input video frames are first preprocessed to obtain the SR images. (**B**) Residual dense blocks are used to extract features from SR images and extract feature information for different dimensions of commodities through the CBAM. (**C**) The feature pyramid network enhances the small commodity region of the shallow feature map, and the fusion of multi-scale feature maps is used to classify the commodities by adaptive regression. Downsample denotes the downsampling operation on the feature map. Upsample denotes the upsampling operation on the feature map.

Figure 1 shows the small commodity detection method employed in this study. The flowchart of the algorithm is divided into three primary steps: (1) preprocessing the input video frame to obtain the SR image; (2) extracting features from SR images and extracting feature information of different dimensions of commodities through the attention module; and (3) enhancing the small commodity area of the shallow feature map in the feature pyramid and classifying the commodities by adaptive regression through the fusion of the multi-scale feature maps.

### 2.1. Video Frame Preprocessing

#### SR Processing

**Figure 2:**
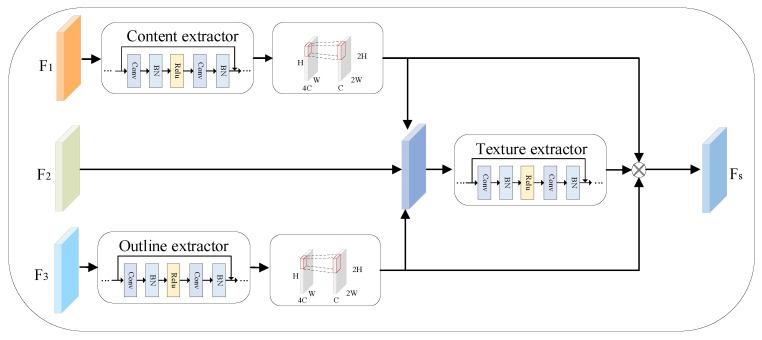
The architecture of image super-resolution (SR) network. $F_{1}$ denotes the content input, $F_{2}$ denotes the texture input, and $F_{3}$ denotes the outline input.

Owing to the low number of pixels in an input video frame, the high-frequency detail information in the image may be substantially missing. This is not conducive to the small commodity detection task. Inspired by the FTT [13], the approach proposed in this study extracts the corresponding semantic features of images through content, texture, and contour extractors and thus improves the resolution of content and texture features to four times those of the original images. Simultaneously, it extracts the contour features of the features extracted from the content. Subsequently, it stitches the content, texture, and contour features together to obtain high-resolution features. Thus, it achieves the goal of using low-resolution images to output high-resolution images. The network structure is illustrated in Figure 2.

The structure consists of three parts corresponding to three functions, namely content extraction, texture extraction, and outline extraction. $F_{1}$ is the content input, $F_{2}$ is the texture input, and $F_{3}$ is the outline input. Herein, sub-pixel convolution was used to perform advanced spatial resolution processing on the input features. Subpixel convolution is a transformation for processing pixels in the channel dimension, whereby $F_{0}\in R^{H\times W\times C\cdot l^{2}}$ is transformed into $F_{0}\in R^{H\cdot l\times W\cdot l\times C}$. $F_{1}$ extracts the image’s semantic features through the content extractor and converts the output multi-channel feature map into a single-channel feature map. $F_{2}$ initially maintains its resolution and then fuses it with $F_{1}$ for the feature map. The semantic features play an important role in SR image restoration; however, the generated video frames lack contour features. Therefore, in this study, an outline extractor was added to represent the contour information. $F_{3}$ was input into the outline extractor to obtain contour features, and the output multi-channel feature map was converted into a single-channel feature map. Then, we input the content feature map. Subsequently, the content feature map, $F_{2}$, and contour feature map were input into the texture extractor for texture feature extraction. The generated feature map contains rich semantic information. Finally, it was stitched with the feature map output using the content and contour extractors to obtain the feature map $F_{s}$ containing the texture, content, and contour information. The expression is as follows:

(1) $$F_{s}=R_{T}(F_{2}⊗(R_{C}(F_{1})↑2\times )⊗R_{O}(F_{3})↑2\times +R_{C}(F_{1})↑2\times +R_{O}(F_{3})↑2\times )$$

where $R_{C}(\cdot )$ is the content extraction module; $R_{O}(\cdot )$ is the outline extraction module; and $R_{T}(\cdot )$ is the texture extraction module.

The original image and detailed feature map were fused, and $F_{low}$ represents the source image features, which were merged with $F_{s}$ through the fusion layer and described as follows:

(2) $$F_{fusion}=H_{Concat}(F_{low}+F_{s})$$

where $H_{Concat}$ denotes the fusion operation. The fusion layer is essentially a bottleneck layer for providing feature fusion and increasing the nonlinear relationship between the high- and low-resolution features. Subsequently, the merged image features are input into the image reconstruction network for high-quality SR image reconstruction [8]. The formula is expressed as follows:

(3) $$I_{SR}=F_{IRN}(F_{fusion})$$

where $F_{IRN}$ stands for image reshaping operation and $I_{SR}$ represents the reconstructed image.

### 2.2. Feature Extraction

In the feature extraction process, a residual network is used for feature extraction. To fully extract the features of commodity regions, an attention mechanism is added to the network to focus on commodity regions and extract fine commodity feature information.

#### 2.2.1. Residual Network

In this study, a residual network comprising multiple residual units stacked together was used. Owing to the general lack of small commodity features, the proposed approach uses residual dense blocks to connect and supplement local features when extracting features and reduces the number of network parameters through parameter sharing. The network adds a skip connection between each residual unit and the next one and fuses the output features of the different residual units. The skip connection in the residual block helps maintain the norm of the gradient and ensures stable backpropagation.

Essentially, the feature map $F^{W\times H\times C\cdot l^{2}}$ is sent to the three channels of the residual network for effective feature extraction. The proposed network was divided into two parts: the residual backbone network and the attention mechanism module [29]. In the backbone network, a 3×3 convolution kernel was used for feature extraction, and a $F_{1},\cdots ,F_{i},\cdots ,F_{n}$ feature map was obtained. The map can be expressed as follows:

(4) $$F_{i}=H_{Conv}(F_{i-1})$$

where $H_{Conv}(\cdot )$ includes the convolution layer, batch normalization (BN) layer, and rectified linear unit (ReLU) function.

The residual dense blocks are fused in each branch to obtain a dense feature map. The corresponding equation is as follows:

(5) $$F_{k+1,C_{t}}=H_{Conv,1\times 1}(H_{Concat}(F_{k+1,C_{1}},\cdots ,F_{k+1,C_{t-1}}))$$

where $H_{Concat}(\cdot )$ represents the feature fusion and $H_{Conv,1\times 1}(\cdot )$ is the convolutional layer, BN layer, and a Relu nonlinear layer.

The output feature map $F_{k+2}$ was obtained by adding the input feature map $F_{k}$ and dense feature map $F_{k+1}$ [36]. The formula is as follows:

(6) $$F_{k+2}=F_{k}+F_{k+1}$$

**Figure 3:**
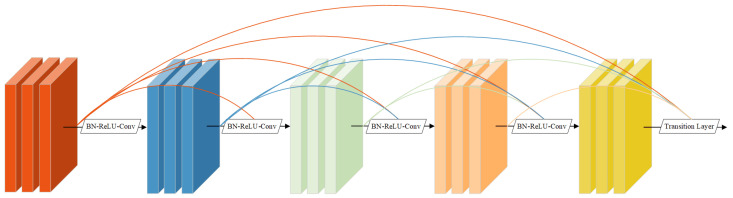
The architecture of the residual network.

The structure of the residual network is shown in Figure 3.

#### 2.2.2. Attention Mechanism Module

**Figure 4:**
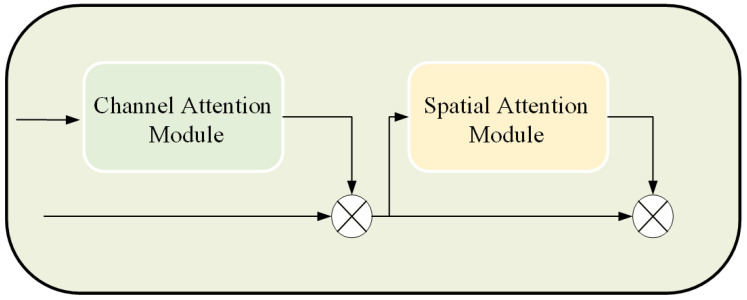
The architecture of the attention mechanism module.

To address the problem of insufficient utilization of features in the middle of the network, CBAM was introduced in the middle of the residual network [29] to enhance its representational ability. To avoid the loss of salient features of small items in the extraction process, an attention mechanism based on both channel and spatial attention was used. A convolution operation was employed to mix the cross-channel and spatial information and extract the important feature information of the small commodities. The structure of the attention mechanism is shown in Figure 4.

The input feature map $F\in R^{C\times H\times W}$, one-dimensional channel attention map $F\in R^{C\times 1\times 1}$, and two-dimensional spatial attention map $M_{s}\in R^{1\times H\times W}$ are described as follows:

(7) $$F^{\prime }=M_{c}(F)⊗F$$

(8) $$F^{\prime \prime }=M_{s}(F^{\prime })⊗F^{\prime }$$

where ⊗ is the pixel-by-pixel multiplication and $F^{\prime \prime }$ is the refined feature of the output.

### 2.3. Feature Fusion and Detection Frame Regression

#### 2.3.1. Feature Pyramid Network

**Figure 5:**
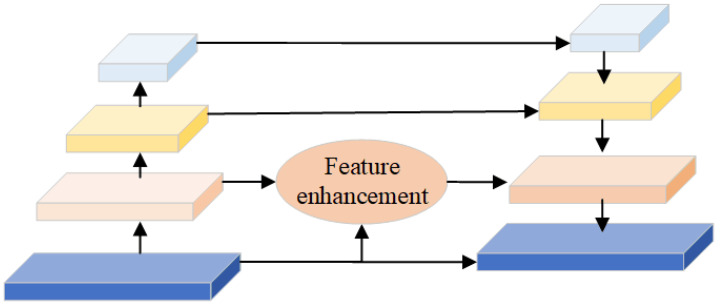
The architecture of the feature pyramid network. The FPN is used to produce multi-scale feature representations.

Existing object detectors have achieved good results for large objects; however, their performance for small objects is unsatisfactory. In this study, to detect smaller commodities, an image FPN was constructed to realize detection across the scale range. In particular, a lightweight architecture that efficiently generates image feature pyramids in the detection framework was used. The structure of the FPN is shown in Figure 5.

The extracted features were sampled to obtain multi-scale feature maps, namely, $G_{1},G_{2},G_{3},G_{4}$. The feature maps of different scales were upsampled and fused to obtain $S_{1},S_{2},S_{3},S_{4}$. This approach can fully utilize different context regions to obtain global information, including high-level semantic and shallow location information. The region proposal network adaptively generates proposal regions and sends them to the subsequent network.

#### 2.3.2. Feature Enhancement

**Figure 6:**
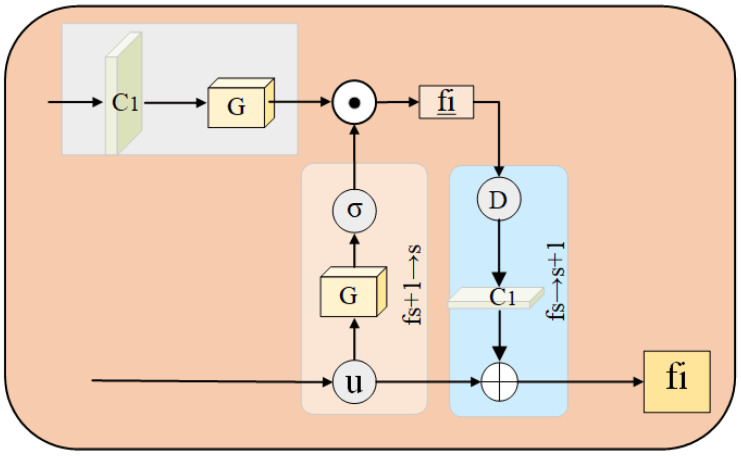
The architecture of the feature enhancement network. $C_{1}$ is the 1×1 convolution kernel.

Small commodities contain less information in the feature maps, and such information can easily be ignored. To efficiently detect small commodities, the salient features of commodities are emphasized and expressed, which is helpful in achieving the rapid detection of commodities. By improving the neighborhood transmission module [32], a feature enhancement network was designed herein. Compared to the deep feature map, the shallow feature map contains richer information regarding the locations, textures, and outlines of commodities. Therefore, the shallow feature map from the FPN was input into the feature enhancement module to enhance the location, contour, and texture information along with other features of small items to improve the detection accuracy and speed performance. The feature enhancement network structure is shown in Figure 6.

The feature enhancement module was used to enhance the features of small commodities, that is, to enhance the features of the shallow feature maps $S_{1}$ and $S_{2}$. First, $S_{1}$ was upsampled. Subsequently, spatial channels were generated using a gate function, and a feature map $S_{1}^{\prime }$ was obtained based on the activation function. The input $S_{2}$ underwent convolution by a 1×1 convolution kernel, and $S_{2}^{\prime }$ was obtained by a gate function operation. By multiplying the features of $S_{1}^{\prime }$ and $S_{2}$, calculations were obtained as follows:

(9) $$S_{1}^{\prime }=\sigma (G(U(S_{1})))=\frac{1}{1+e^{-G(U(S_{1}))}}$$

(10) $$S_{2}^{\prime }=G(H_{Conv,1\times 1}(S_{2}))$$

where σ(·) is the activation function; U(·) is the upsampling operation on the feature map; and G(·) is the self-attention gate function, which generates a spatial channel to enhance commodity features. The formula is as follows:

(11) $$G(S_{i})=H_{Conv}(S_{i})$$

The combination of the two results in $S_{p}$, which can be expressed as follows:

(12) $$S_{p}=S_{1}^{\prime }⊙S_{2}^{\prime }$$

where ⊙ denotes the Hadamard product. These features were summed element-by-element to obtain the details $\underline{S_{k}}$, as follows:

(13) $$\underline{S_{k}}=S_{1}⊕H_{Conv,1\times 1}(D(S_{p}))$$

where ⊕ stands for a pixel-by-pixel summation; D(·) is the downsampling operation on the feature map; and the detailed features of the commodity area are enhanced to facilitate subsequent classification and detection box regression. Thus, enhanced feature maps $\underline{S_{k}}$ and high-level feature maps $S_{1}^{\prime }$, $S_{3}^{\prime }$, and $S_{4}^{\prime }$, containing the location information, contour information and center point of the commodity, were obtained. These can be used to effectively predict different scales and to generate subsequent product detection frames.

#### 2.3.3. Commodity Detection Frame

As our task was to generate a commodity detection box, a region proposal network was introduced for commodity region regression. In the training phase, 10,000 regression boxes with the highest scores were obtained through a non-maximum suppression operation, and 1500 of them were selected as small-item proposals. In the test phase, 400 proposals were selected from 10,000 regression frames. Owing to the occlusion of small commodities and relatively few features, detection in regression detection frames can be easily missed. Therefore, inspired by a previous study [37], a new loss function was proposed to train the network. The loss function in this article consists of three parts. The first part is the regression loss function, which has a great influence on the regression of the detection box owing to the variety of shapes of the commodities. To solve this problem, the intersection-over-union (IOU) factor $\frac{\left|-\log(IOU)\right|}{\left|L_{1}(v_{kj}^{\prime },v_{kj})\right|}$ was introduced to optimize positioning accuracy and accurately return the detection box for commodities. The formula is as follows:

(14) $$L_{1}(x)=\left\{\begin{array}{c} 0.5x^{2},\left|x\right|<1\\ \left|x\right|-0.5,otherwise \end{array}\right.$$

(15) $$L_{reg}=\frac{1}{N}\sum_{i=1}^{N}\sum_{j\in \left\{x,y,w,h\right\}}\frac{L_{1}(v_{kj}^{\prime },v_{kj})}{\left|L_{1}(v_{kj}^{\prime },v_{kj})\right|}\left|-\log(IOU)\right|$$

where $L_{1}$ is the smoothing loss; N is the number of regression frames; and IOU represents the overlap between prediction frames and real frames.

By regressing the size of the target commodity *c*, the regressed commodity width and height were obtained as $S_{c}=(x_{2}^{\left(c\right)}-x_{1}^{\left(c\right)},y_{2}^{\left(c\right)}-y_{1}^{\left(c\right)})$. The second part is a loss function for the commodity width and height. The loss function was used to measure the losses of commodity width and height. The formula is described as follows:

(16) $$L_{h,w}=\frac{1}{N^{2}}{\sum_{i=1}^{N}\left|{\overset{⏜}{S}}_{real}-S_{c}\right|}^{2}$$

${\overset{⏜}{S}}_{real}$ is the true width and height of the commodity.

The third part is the classification loss function, which comprises the cross-entropy function, and is expressed as follows:

(17) $$L_{cls}=\frac{1}{N}\sum_{i=1}^{N}\log\frac{e^{W_{C_{i}}^{T}x_{i}+b_{C_{i}}}}{\sum_{i=1}^{N}e^{W_{j}^{T}x_{i}+b_{j}}}$$

where $W_{C_{i}}^{T}$ is the learned weight; and $b_{j}$ is the bias term.

The formula for the total loss function is defined as follows:

(18) $$L_{total}=\lambda_{1}L_{reg}+\lambda_{2}L_{h,w}+\lambda_{3}L_{cls}$$

Among them, the distribution of the super-parameters, $\lambda_{1}=\frac{1}{3}$, $\lambda_{2}=\frac{1}{3}$, $\lambda_{3}=\frac{1}{3}$ controls the weight of each loss function. Through the constraint of the loss function, an accurate detection frame was derived, thereby completing the detection task for small commodities.

## 3. Experiments

**Figure 7:**
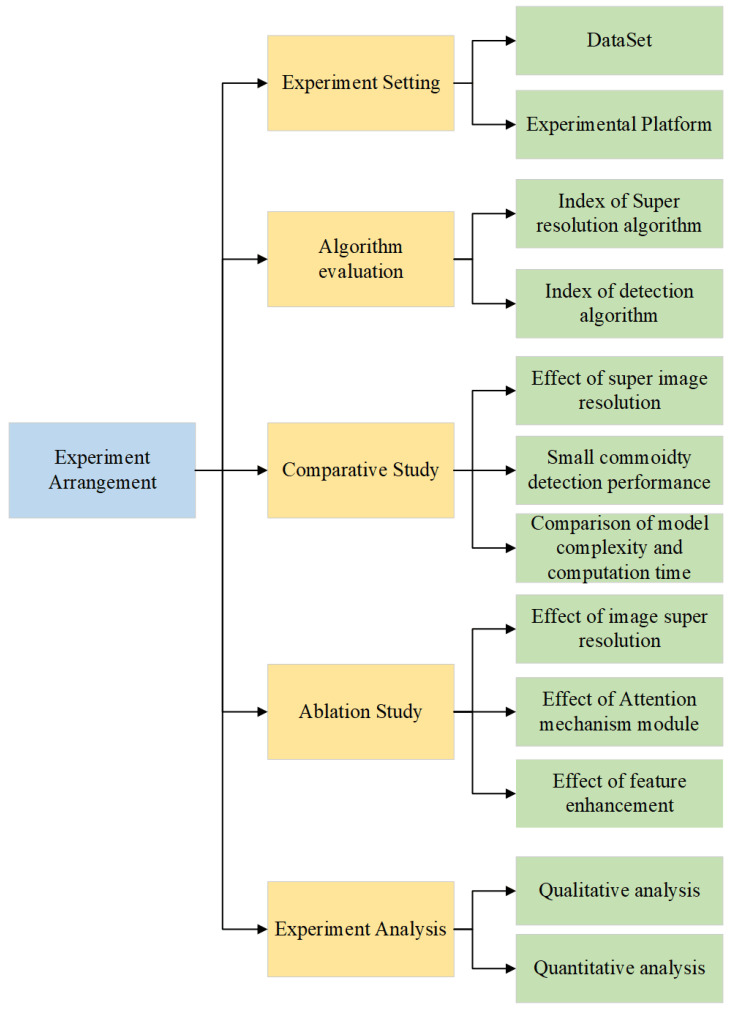
Overview of experiment arrangement.

The specific structure of the experimental content in this study is shown in Figure 7 and is mainly divided into five parts: experimental setting, algorithm evaluation, comparison study, ablation study, and experimental analysis.

### 3.1. Experiment Setting

#### 3.1.1. Dataset

In this study, self-made retail containers were used to collect 16 commodity datasets, including training, validation, and test sets. Herein, the definition of the small commodity is the size of the small commodity, which is related to the size of a human hand. In particular, under extreme conditions, whether the entire hand of a consumer can fully cover the features of the effective area of the commodity to the greatest extent such that the commodity cannot be detected was determined. Commodities meeting this condition were considered small commodities.

To facilitate the subsequent commodity inspection, the names of the commodities were simplified, as shown in Table 1.

**Figure 8:**
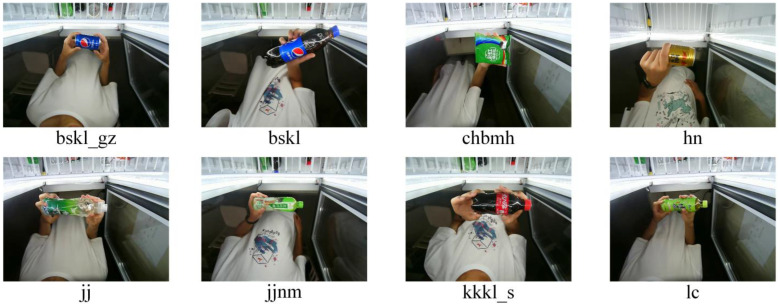

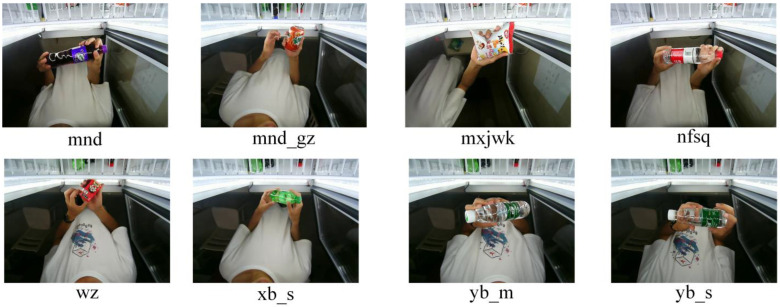
Sample datasets of each commodity.

**Figure 9:**
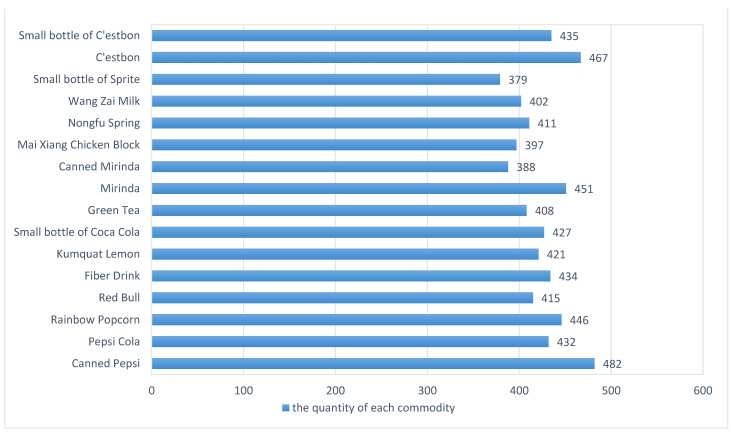
Quantity distribution of each commodity.

To illustrate the feasibility of the experimental data, commodity datasets were collected under appropriate lighting conditions. The datasets for each commodity, including both large and small commodities, are shown in Figure 8 and Figure 9.

#### 3.1.2. Experimental Platform

In this work, the system platform was Windows 10, the GPU model was an NVIDIA GeForce RTX 3060, the CPU was an I5-12400F, the memory was 16 GB, and the software environment was Python3.7 and Pytorch2.3.

### 3.2. Algorithm Evaluation

#### 3.2.1. Index of SR Algorithm

To illustrate the processing results from the SR algorithm, two quantitative indicators, namely the peak signal-to-noise ratio (*PSNR*) and structural similarity measure (*SSIM*), were introduced. The *PSNR* formula is expressed as follows:

(19) $$PSNR=20\times \log_{10}(\frac{MAX_{I}}{MSE})$$

where $MAX_{I}$ represents the maximum pixel value in the image pixels; and MSE represents the mean square error of the corresponding pixels between the generated image $f_{ij}^{\prime }$ and original image $f_{ij}$. MSE is calculated as follows:

(20) $$MSE=\frac{1}{M*N}\sum_{i=1}^{N}\sum_{j=1}^{M}{\left(f_{ij}-f_{ij}^{\prime }\right)}^{2}$$

*SSIM* is a measure of the similarity between two images, and it is calculated as follows:

(21) $$SSIM(x,y)=\frac{2\mu_{X}\mu_{Y}+c_{1}}{\mu_{X}^{2}+\mu_{Y}^{2}+c_{1}}*\frac{2\sigma_{X}\sigma_{Y}+c_{2}}{\sigma_{X}^{2}+\sigma_{Y}^{2}+c_{2}}$$

where, $\mu_{X}$ and $\mu_{Y}$ are the pixel mean of image *X* and image *Y*, respectively; $\sigma_{X}$ and $\sigma_{Y}$ are the pixel variances of image *X* and image *Y*, respectively; $c_{1}={\left(0.01*l\right)}^{2}$; and $c_{2}={\left(0.03*l\right)}^{2}$.

Note that the higher the *PSNR*, the less distorted the processed image is. A higher *SSIM* indicates higher image similarity and better image quality.

#### 3.2.2. Index of Detection Algorithm

To evaluate the proposed algorithm, the average precision (*AP*) and mean AP (*mAP*) were selected as evaluation indicators.

(22) $$Accuracy=\frac{TP+TN}{TP+TN+FP+FN}$$

(23) $$Precision=\frac{TP}{TP+FP}$$

(24) $$Recall=\frac{TP}{TP+FN}$$

(25) $$F_{1}-score=2\frac{Precise\cdot Recall}{Precise+Recall}$$

(26) $$AP_{i}=\frac{1}{i}\sum P\cdot dr$$

(27) $$mAP=\frac{\sum_{i}^{N}AP_{i}}{N}$$

where *TP* denotes a positive sample and a positive prediction result; *TN* denotes a positive sample and a negative prediction result; *FP* denotes a negative sample and a positive prediction result; and *FN* denotes a negative sample and negative prediction result; *Accuracy* represents the proportion of all correct predictions. *Precision* represents the percentage of true positive predictions; *Recall* denotes the proportion of true positives; *AP* is the area of the *Precision-Recall* curve; and *mAP* is the mean average accuracy across all classes.

### 3.3. Comparative Study

**Figure 10:**
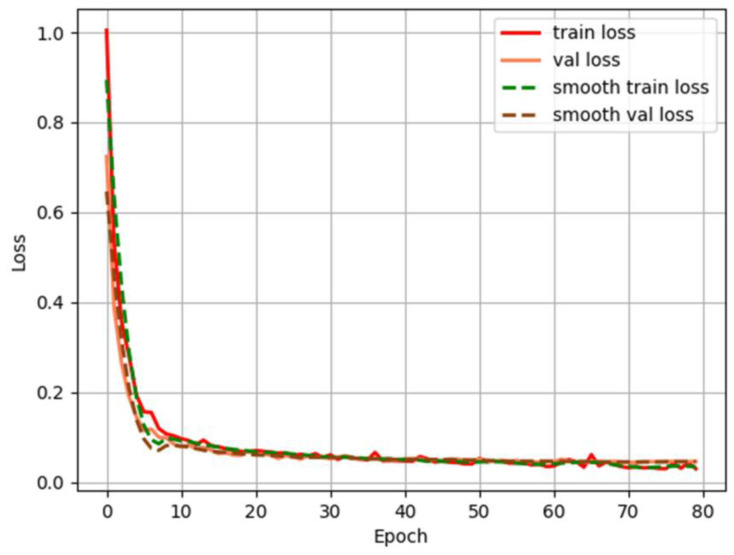
Loss function curve.

The algorithm proposed herein has good completeness. The relevant parameters were set to achieve high-performance small commodity detection. The total training batch was 100, the epoch was 80, and the learning rate was set to 0.0001. The loss function curve is shown in Figure 10.

As can be seen from the figure, when the training reached approximately 80 iterations, the loss function converged.

#### 3.3.1. Effect of SR

To demonstrate the effectiveness of the SR method, it was compared to the SRGAN [38], EDSR [39], and CARN [40] algorithms in the context of image blurring during the detection process. The abovementioned experiments revealed that the image details processed by the proposed method were richer, and the generated image had a high degree of similarity to the original image. Table 2 lists the experimental results obtained by the different methods.

Compared to the other methods, the method proposed herein was superior in terms of index performance. The *PSNR* and *SSIM* values were the highest. The SR images significantly restored the details of the original image, and the original image features were retained to the greatest extent, thus demonstrating the feasibility and superiority of this algorithm.

**Figure 11:**
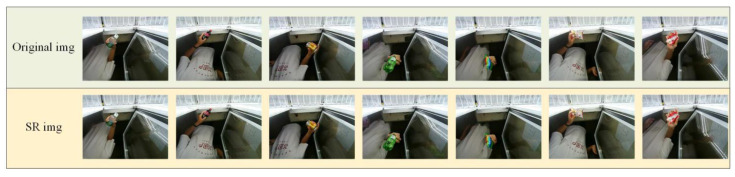
Comparison of original images and SR-processed images.

The experimental results are shown in Figure 11, with original images (a) and (c) and SR images (b) and (d) obtained by the proposed method.

Evidently, from the above figure, the SR processing retained the texture and structure information of the original image and enhanced the high-frequency information, such as the commodity contents and contours in the image. The semantic information of commodities was restored, thereby avoiding the problem of image distortion (which led to a decline in detection accuracy).

The SR processing not only retains the original image but also restores the commodity contour and other features to a certain extent, thereby playing a positive role in the subsequent commodity contour feature extraction.

#### 3.3.2. Small Commodity Detection Performance

When consumers buy commodities, the camera captures different degrees of occlusion of the commodities from its perspective, which can considerably affect the effects of small-object detection. To verify the efficiency of this algorithm, the model was compared with four other algorithms, and four types of commodities with different degrees of occlusion were selected. These included slight occlusions (occlusion degrees of 0–10%), partial occlusions (occlusion degrees of 10–20%), moderate occlusions (occlusion degrees of 20–30%), and severe occlusions (occlusion degrees of 30–60%). The experimental results are listed in Table 3.

According to the above table, the proposed method was superior to the SSD, Faster-RCNN, Yolov5, and RetinaNet algorithms in terms of detection accuracy on commodity datasets with different degrees of occlusion. The horizontal comparison indicates that the proposed method performed well in terms of detection accuracy for different commodities. Under severe occlusion, the detection accuracy of the proposed algorithm was improved by more than 3% relative to the mainstream methods Yolov5 and RetinaNet. The longitudinal comparison indicated that with an increase in the degree of occlusion, detection accuracy exhibited a gradual downward trend. Compared with other algorithms, the detection accuracy of the proposed method generally remained above 81%, and the proposed model was relatively stable compared with the other models. In addition, the results demonstrated the superiority of the proposed algorithm. In terms of detection speed, the proposed model reached an average of 15.13 frames/s, thus meeting the requirements for real-time performance.

#### 3.3.3. Comparison of Model Complexity and Computation Time

To evaluate the computational complexity of each model, relevant comparison experiments were performed in terms of the number of model parameters and training time, and the results are shown in Table 4. The time indicates the training time. As can be seen from the table, the Faster R-CNN model has the largest number of parameters, while the RetinaNet model has a relatively small number of parameters. Compared with other models, the number of parameters and training time of this paper need to be further reduced, and the complexity of the model needs to be optimized to meet the commercialization requirements.

### 3.4. Ablation Study

#### 3.4.1. Effect of SR

The comparative experiments revealed that the quality of the image generated by the SR algorithm was high and that the contours of the commodities and other information were significantly restored. To explore whether SR commodity detection was efficient, ablation experiments were performed under two conditions: (1) lack of SR commodity detection and (2) commodity detection under SR. The experimental results are listed in Table 5.

The image detection effect after SR processing was significantly higher than that of the original image. In terms of detection accuracy, the performance with SR processing was better than that without SR processing, with an increase of more than 9%. The SR-processed image contour feature information was more abundant, the network could further extract the semantic details of small commodities, and the small commodity detection accuracy was significantly improved, further verifying the effectiveness of the SR algorithm.

#### 3.4.2. Effect of Attention Mechanism Module

**Figure 12:**
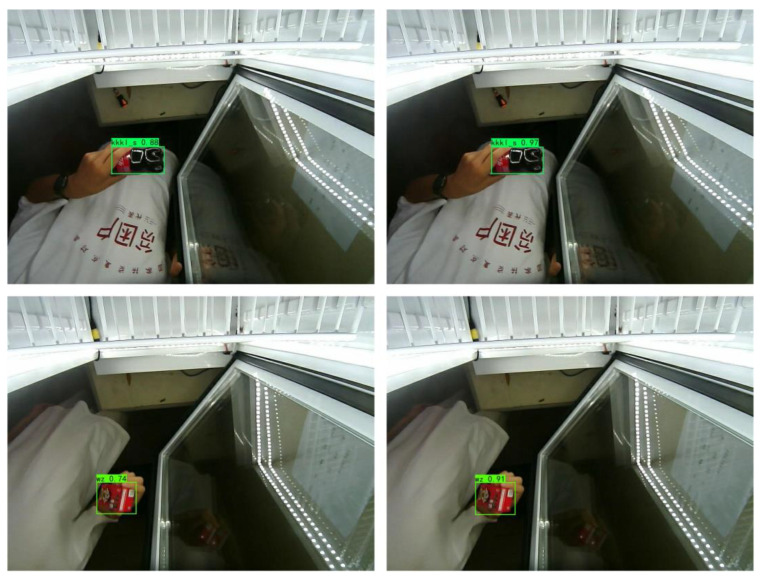
Detection results with and without the attention mechanism.

Insufficient information extraction from small commodities can easily occur in the feature extraction process. This study focuses on commodity feature information using an attention mechanism. To verify the effectiveness of the method, experiments on feature extraction with an attention mechanism were conducted. The results of the commodity detection are shown in Figure 12.

According to the above experimental results, the detection accuracy of the right figure was significantly improved compared to that of the left figure, which indicates that the small commodity detection effect was significantly improved under the effect of the attention mechanism.

**Figure 13:**
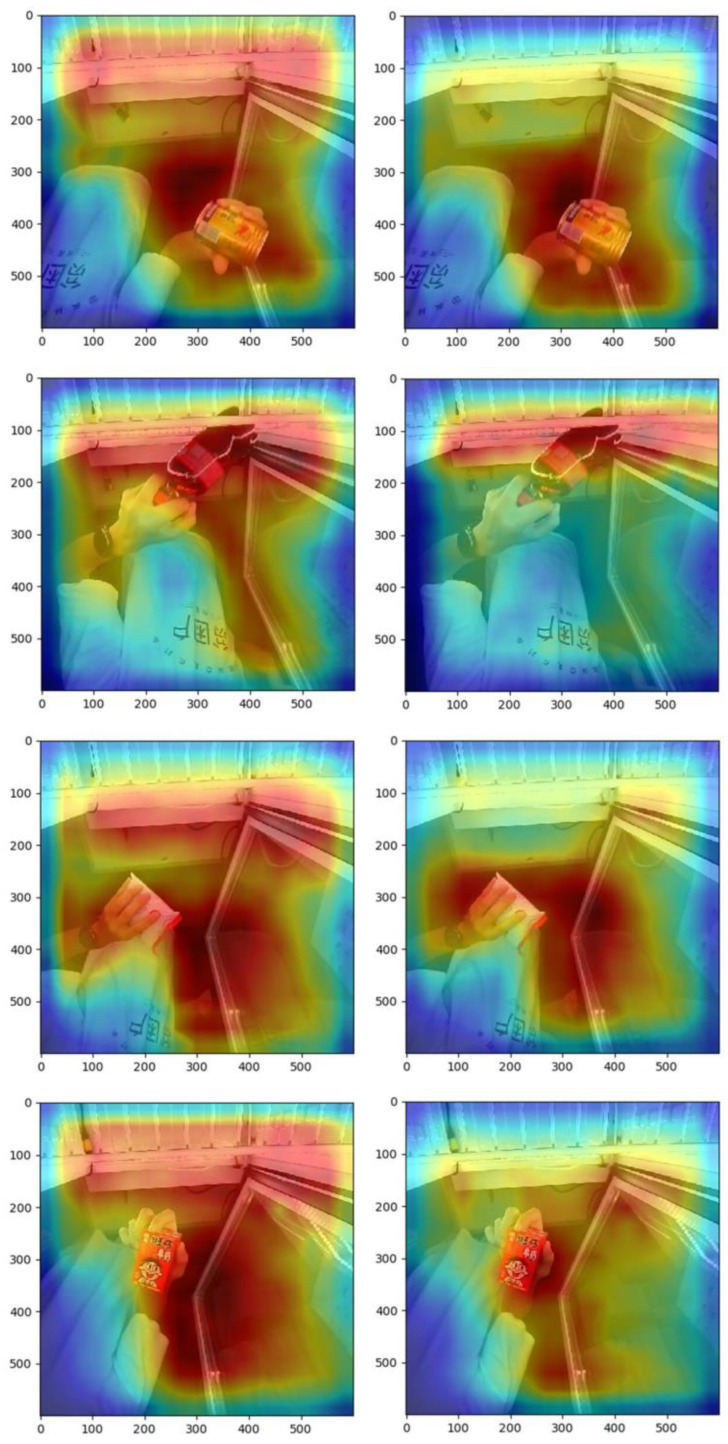
Heatmaps with and without the attentional mechanism.

To understand which parts of the network were focused on based on the attention mechanism, the feature map of the feature extraction part could be visualized through heatmaps. The size of the output feature maps was set to 600 × 600 in this experiment, as shown in Figure 13.

The left and right panels, respectively, show heatmaps without and with the attention mechanism. Evidently, under the effect of the attention mechanism, the network focus area is significantly reduced, and the network extracts more refined commodity regional features from the channel and spatial dimensions, improving the efficiency of commodity feature extraction to a certain extent. In addition, it further verifies the feasibility and scientificity of using the attention mechanism in the network.

#### 3.4.3. Effect of Feature Enhancement

**Figure 14:**
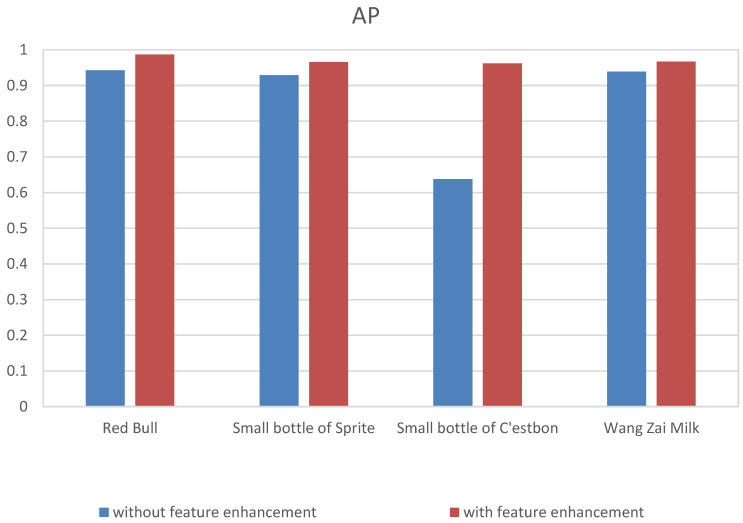
Ablation experiment of detection results with and without the feature enhancement.

To evaluate the effect of the feature enhancement module on the detection of occluded small commodities, experiments were conducted using both an ordinary FPN and an FPN with a feature enhancement module (FPN + FEM). The experimental results are shown in Figure 14.

The commodity detection accuracy after adding the feature enhancement could reach approximately 96%, and the highest accuracy reached was 98%. Compared with direct prediction, it had a higher accuracy. Simultaneously, for the small bottles of Sprite with fewer effective features, the accuracy was increased by 32.4% relative to the original, and the detection effect was more significant.

**Figure 15:**
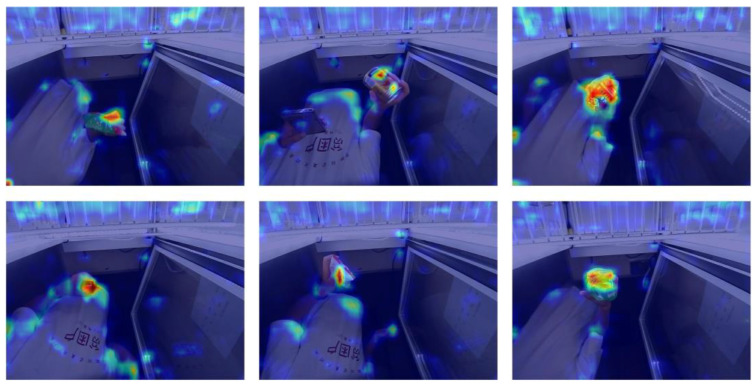
Feature-enhanced visualization.

Figure 15 shows the results for the commodity feature region after feature enhancement. According to the figure, the important features of the commodity were enhanced, and the detection performance of the network was further improved, thus verifying the effectiveness of the feature enhancement module proposed in this study.

### 3.5. Experiment Analysis

#### 3.5.1. Qualitative Analysis

**Figure 16:**
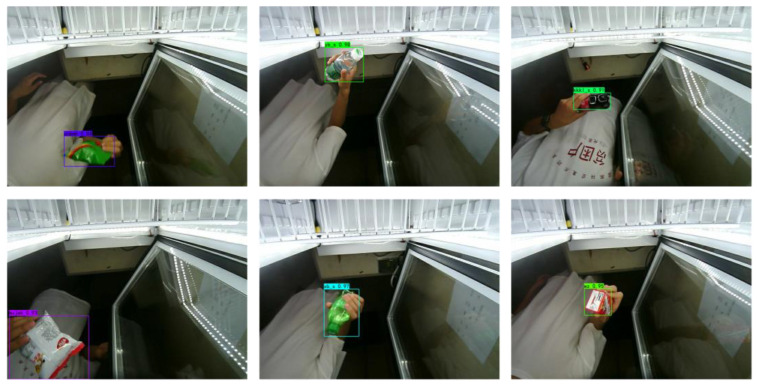
Detection results for different types of small commodities.

In this study, different types of small commodities with different degrees of occlusion were selected to analyze the proposed algorithm, and the results are shown in Figure 16.

**Figure 17:**
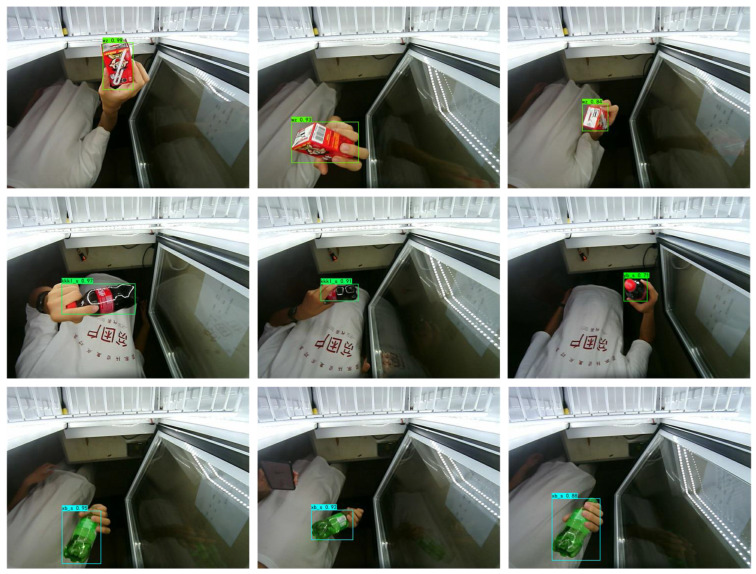
Detection results for small commodities with different degrees of occlusion.

The results revealed excellent performance in the detection of small commodities of different types and occlusion levels. As shown in Figure 17, the detection performance of the network was stable under different occlusion levels, and the detection accuracy of the commodities still reached as high as 80% in the case of severe occlusion. This method increased the high-frequency information of the image through SR; simultaneously, the feature enhancement module in the FPN could effectively improve the feature expression of small commodities, and therefore, the detection effect was significantly improved. The detection results qualitatively illustrate the feasibility and efficiency of the algorithm used in this study.

#### 3.5.2. Quantitative Analysis

To illustrate the commodity detection performance of the proposed algorithm, a quantitative comparison was performed with five mainstream networks, and Table 6 lists the commodity detection results of the different algorithms.

According to the above table, the different algorithms achieved good detection performance. The algorithm in this study achieved excellent performance in terms of the F1-score and mAP indicators, with an F1-score of 0.983 and an mAP of 0.9847, which are superior to the results for other algorithms. The algorithm network in this study comprised Resent50 + CBAM + FPN (FAM). Compared to RetinaNet, the F1-score improved by 2.6 % and the mAP improved by 2.45%.

To illustrate the detection accuracy of the algorithm, different lightly occluded commodity data were selected for comparative experiments. The results are presented in Table 7.

The detection accuracies for different commodity categories varied considerably. Because the features of Red Bull were more significant, each network had high detection accuracy. Compared with YOLOv5, the overall accuracy of this method was higher. The proposed method had a detection effect on different small commodities. The detection performance of the model was more stable than that of the other methods while maintaining accuracy.

## 4. Conclusions

In this article, a local adaptive feature enhancement detection algorithm for occluded small commodities under super-resolution is proposed. Based on the low image clarity, a new SR algorithm is designed that effectively improves the image clarity by adding contour features to the feature texture transmission module, fusing them with texture and content features, obtaining rich fine features, and obtaining high-frequency image information through reconstruction. To effectively express small commodities occluded in complex environments, a self-attention gate function is used to generate commodity space channels, enhance commodity texture features, and other characteristics, and further improve the detection accuracy of small commodities. Experimental results show that the proposed algorithm has good detection accuracy and can effectively reduce the false or missed detections caused by complex occlusion. However, the method in this article pursues commodity detection accuracy and ignores the light weight of the model, which considerably limits the detection speed of the model. In the future, the network model will be further explored and optimized to reduce the number of model parameters and achieve real-time detection of small commodities.

## Figures and Tables

**Table 1:** The simplified table of commodity names.

Name of Commodity	Simplification of Commodity Name
Canned Pepsi	bskl_gz
Pepsi Cola	bskl
Rainbow Popcorn	chbmh
Red Bull	hn
Fiber Drink	jj
Kumquat Lemon	jjnm
Small bottle of Coca Cola	kkkl_s
Green Tea	lc
Mirinda	mnd
Canned Mirinda	mnd_gz
Mai Xiang Chicken Flavor Block	mxjwk
Nongfu Spring	nfsq
Wang Zai Milk	wz
Small bottle of Sprite	xb_s
C’estbon	yb_m
Small bottle of C’estbon	yb_s

**Table 2:** Super-resolution results of different algorithms.

Method	SRGAN	EDSR	CARN	Ours
*PSNR*/dB	28.16	30.46	32.12	**32.72**
*SSIM*	0.889	0.887	0.858	**0.894**

Note: Bold is the best result.

**Table 3:** Comparison of detection performance of different algorithm models.

Occlusion Degree of Different Commodity	SSD	Faster-RCNN	YOLOv5	RetinaNet	Ours
Red Bull (slight occlusion)	0.9667	0.9693	0.9682	0.9883	**0.9891**
Red Bull (partial occlusion)	0.9430	0.9451	**0.9671**	0.9554	0.9557
Red Bull (moderate occlusion)	0.8747	0.8366	0.8964	0.9189	**0.9273**
Red Bull (heavy occlusion)	0.6746	0.7529	0.8153	0.8482	**0.8828**
C’estbon (slight occlusion)	0.6758	0.9833	0.9634	0.9839	**0.9857**
C’estbon (partial occlusion)	0.5693	0.9408	0.9567	0.9673	**0.9724**
C’estbon (moderate occlusion)	0.5351	0.8021	0.8467	0.8017	**0.9152**
C’estbon (heavy occlusion)	0.4332	0.7475	0.7676	0.7631	**0.8281**
Sprite (slight occlusion)	0.8807	0.8863	0.9757	0.9603	**0.9787**
Sprite (partial occlusion)	0.8356	0.8525	0.9363	0.8592	**0.9576**
Sprite (moderate occlusion)	0.7221	0.7889	0.8485	0.7975	**0.8972**
Sprite (heavy occlusion)	0.4533	0.7218	0.7227	0.7919	**0.8483**
Wang Zai Milk (slight occlusion)	0.7507	0.9163	0.9574	0.9752	**0.9793**
Wang Zai Milk (partial occlusion)	0.7436	0.8011	0.9369	0.9239	**0.9679**
Wang Zai Milk (moderate occlusion)	0.6492	0.6125	0.7495	0.8702	**0.9362**
Wang Zai Milk (heavy occlusion)	0.4750	0.5382	0.6756	0.7647	**0.8125** ^1^

Note: Bold is the best result. ^1^ All results are the *AP*.

**Table 4:** Comparison of model complexity.

Model	Backbone	Parameters (M)	Time (min)
YOLOv4	CSPDarknet53	42.3	977
YOLOv5	CSPDarknet53	38.4	854
SSD	VGG16	139.7	3063
Faster R-CNN	VGG16	148.4	3368
RetinaNet	Resnet50	27.5	617
Ours	Resnet50	41.1	918

**Table 5:** Detection accuracy of original image and super-resolution.

Method	The Lack of SR Commodity Detection	Commodity Detection under SR
Mai Xiang Chicken Flavor Block	0.7927	0.8938
Small bottle of C’estbon	0.8083	0.9673
Wang Zai Milk	0.8692	0.9651
Rainbow Popcorn	0.7743	0.9081
Small bottle of Coca Cola	0.7357	0.8635 ^1^

^1^ All results are the *AP*.

**Table 6:** Comparison of the accuracy of commodity detection with different algorithms.

Method	Backbone	F1-Score	*mAP*
YOLOv4	CSPDarknet53	0.932	0.9544
YOLOv5	CSPDarknet53	0.972	0.9740
SSD	VGG	0.979	0.9784
Faster R-CNN	VGG	0.952	0.9768
RetinaNet	Resnet50	0.957	0.9602
YOLOX [41]	CSPDarknet53	0.974	0.9831
DETR [42]	Resnet50	0.980	0.9753
Ours	Resnet50	**0.983**	**0.9847**

Note: Bold is the best result.

**Table 7:** The detection results of different commodity types.

Category	SSD	Faster R-CNN	RetinaNet	YOLOv5	Ours
Coca Cola	0.4409	0.5573	0.5531	**0.8865**	0.8537
Wang Zai milk	0.6807	0.9156	0.9015	0.9332	**0.9671**
Small bottle of Sprite	0.8784	0.9487	0.9495	0.8856	**0.9635**
Small bottle of C’estbon	0..6983	0.9435	0.9647	0.9560	**0.9676**
Red Bull	0.9534	0.9643	0.9779	0.9783	**0.9883**
Canned Mirinda	0.7606	0.9311	0.9353	0.9240	**0.9512** ^1^

Note: Bold is the best result. ^1^ All results are the *AP*.

## Data Availability

Data sharing is not applicable.
